# Supplementary material for: Ecophysiology and niche differentiation of three genera of polyphosphate-accumulating bacteria in a full-scale wastewater treatment plant
Source: mSystems. 2025 Aug 15;10(9):e00322-25. doi: 10.1128/msystems.00322-25 (PMC12455942; doi:10.1128/msystems.00322-25)
Supplement: File S1 — Supplemental figures and tables. [file msystems.00322-25-s0001.pdf]

Supplementary file 1

Ecophysiology and niche differentiation of three genera of polyphosphate accumulating bacteria in a full-scale wastewater treatment plant

Kondrotaite, Z., Petersen, J., Singleton, C., Peces, M., Petriglieri, F., Jensen, T. B. N., Sereika, M., Daugberg A. O. H., Wagner, M., Dueholm, M. K. D., and Nielsen, P. H.

Center for Microbial Communities, Department of Chemistry and Bioscience, Aalborg University, Aalborg, Denmark

1. Methods

1.1 Sampling in full-scale WWTP and short incubations

Fresh activated sludge samples (approx. 4 gSS/L) were aerated for 30 min to remove easily consumable organics and to exhaust some intracellular carbon reserves. After aeration, sludge was transferred to 200 mL serum bottles and sealed with rubber stoppers and aluminum cap. Pure nitrogen was used to flush the headspace of each bottle to ensure anaerobic conditions. In total, 11 different experiments were conducted to investigate the metabolic characteristics of activated sludge microbial communities, using different carbon sources under anoxic conditions, and testing different electron acceptors under anoxic-oxic conditions.

Five different carbon sources (acetate, glucose, amino acid mix, and oleic acid (oleic acid was heated at 60 and dissolved in detergent (0.02 mL Triton X100 in 100 mL oleic acid)) were tested under anoxic conditions. 200 mg COD/L of each carbon source was added to activated sludge. In parallel, control perturbation was performed with no carbon source. Experiments were run under anoxic conditions for a total of five hours. Samples for metatranscriptomics and phosphate measurements were taken at 5 different time points: 20 min. before experiment started (T-1), just after adding the carbon source (T0), 1, 2, and 3 h after carbon source was added.

To test metabolic characteristics with different electron acceptors under anoxic-oxic conditions, a mix of acetate, glucose, and amino acid mix was used as a carbon source under anoxic conditions (200 mg COD/L concentration each). After four hours of anoxic substrate consumption and storage polymer formation, the four different electron acceptors (oxygen, nitrate, nitrite, or nitrous oxide) were added. Samples were collected at 5 different time points: 20 min before the experiment was started, just after adding the carbon mix (T-1), before adding the electron acceptor (T0) and 5, 6 and 7 hours after the carbon source was added.

In both batch experiments, at each sampling point ~2 mL of sample was snap frozen in liquid nitrogen for metatranscriptomics and 4 mL of sample was filtered with 0.22 µm PES filter to measure phosphate (all measurements were performed in triplicates).

1.2 Metagenomes

The DNA was extracted using DNaeasy PowerSoil Pro Kit (Qiagen, Germany) following the manufacturer's recommendations. DNA concentration and quality were checked using Agilent TapeStation genomic DNA screen tapes, Qubit 3.0 fluorometer (Thermo Fisher Scientific, MA, USA), and Nanodrop ND1000 (Thermo Fisher Scientific, MA, USA). After size selection, DNA was stored in TE buffer (pH 8).

DNA libraries for Nanopore sequencing were prepared using the SQK-LSK110 Ligation Sequencing kit (Oxford Nanopore Technologies, UK) according to the manufacturer's instructions. The libraries were sequenced with R9.4.1 chemistry flow cells using a GridION sequencing device.

Short read sequencing libraries were prepared using the Illumina DNA Prep kit in combination with IDT UD Indexes Set A according to the manufacturer's instructions. Final libraries were quantified and insert size evaluated using the Qubit dsDNA HS assay and a D1000 screentape. Samples were multiplexed with the final pooled library created using 100 ng per sample and evaluated in the same way as individual libraries.

The sequenced Nanopore reads were assembled using metaFlye v2.9.1, polished with Racon v1.5.0 (3x rounds) and Medaka v1.6.1 (2x rounds), followed by polishing with Illumina reads using Racon v1.5.0. Automated contig binning was performed using the ensemble method with Metabat2 v2.15, MaxBin2 v2.2.7, Vamb v3.0.7, and Metabinner v1.4.3, while Das Tool v1.1.3 was used to generate the final refined metagenomic bins. To improve genome recovery, coverage values from multiple Illumina read datasets were used as input for the binners.

Table S1. Information about the samples used for metagenomes. The main sample collected from the experiment is in bold. All samples were collected from the aeration tank.

No.	Plant	Date	Date of DNA extraction	Conc. (Qbit)	260/280	260/230
1	AAW	2020 09 16	2022 06 29	166,4	1,92	1,56
2	AAW	2020 11 25	2022 06 29	194,4	1,93	1,53
3	AAW	2021 01 26	2022 06 29	187,2	1,93	0,91
4	AAW	2021 03 24	2022 06 29	224	1,93	1,25
5	AAW	2021 05 05	2022 06 29	168	1,93	1,48
6	AAW	2021 07 21	2022 06 29	168	1,95	0,91
7	AAW	2021 09 23	2022 06 29	132	1,93	1,39
8	AAW	2021 11 24	2022 06 29	110,4	1,94	0,52
9	AAW	2022 01 19	2022 06 29	307,2	1,93	1,64
10	AAW	2022 09 02	2022 06 29	167,2	1,94	1,87
**11**	**AAW**	**2021 11 04**	**2022 06 29**	**230,4**	**1,92**	**1,08**

1.3 Metatranscriptomic data processing

The samples were sequenced on a NovaSeq 6000 using 300 cycles V1.5 S4 flow cell and reagents (20028312). Sample sheets were created with Illumina Experiment Manager V1.19.1 and fastq files created with bcl2fastq2 V2.20.2. The raw sequencing PE reads were filtered and quality-trimmed using fastp V0.23.2 (Chen et al., 2018), GNU parallel (TANGE, 2018) and pigz. Quality trimming was done using the following settings: a minimum length of 50 pb, and average phred quality score of 30, a sliding window of 4 bases with phred score above 20 from the right-hand side of the reads, overlap correlation, deduplication set to 6 and automatic detection of adapters for PE reads. rRNA was removed using RiboDetector v0.2.6 (Deng et al., 2022) using the "-e norma" flag along with "-l 100" and "-shunk_size 256". tRNA, tmRNA and residual rRNA were removed using SortMeRNA v4.34 (Kopylova et al., 2012). CoverM v0.6.0 was used to map the mRNA after SortMeRNA to the

HQ MAG database using 95% identity and 75% alignment. CoverM “filter” was used to subset the bam files so only the reads that passed the alignment and identity thresholds were kept. SAMtools was used to sort the bam files by name using “samtools sort -n” and the fastq reads were extracted from these bam files using “samtools fastq -F 0x4”. Consequently, mRNA reads with at least 95% sequence identity and at least 75% alignment to the MAGs were filtered and kept for the further analysis. This was done to ensure stringent mapping and reduce the likelihood of transcripts from other species mapping to the MAGs.

2 Results

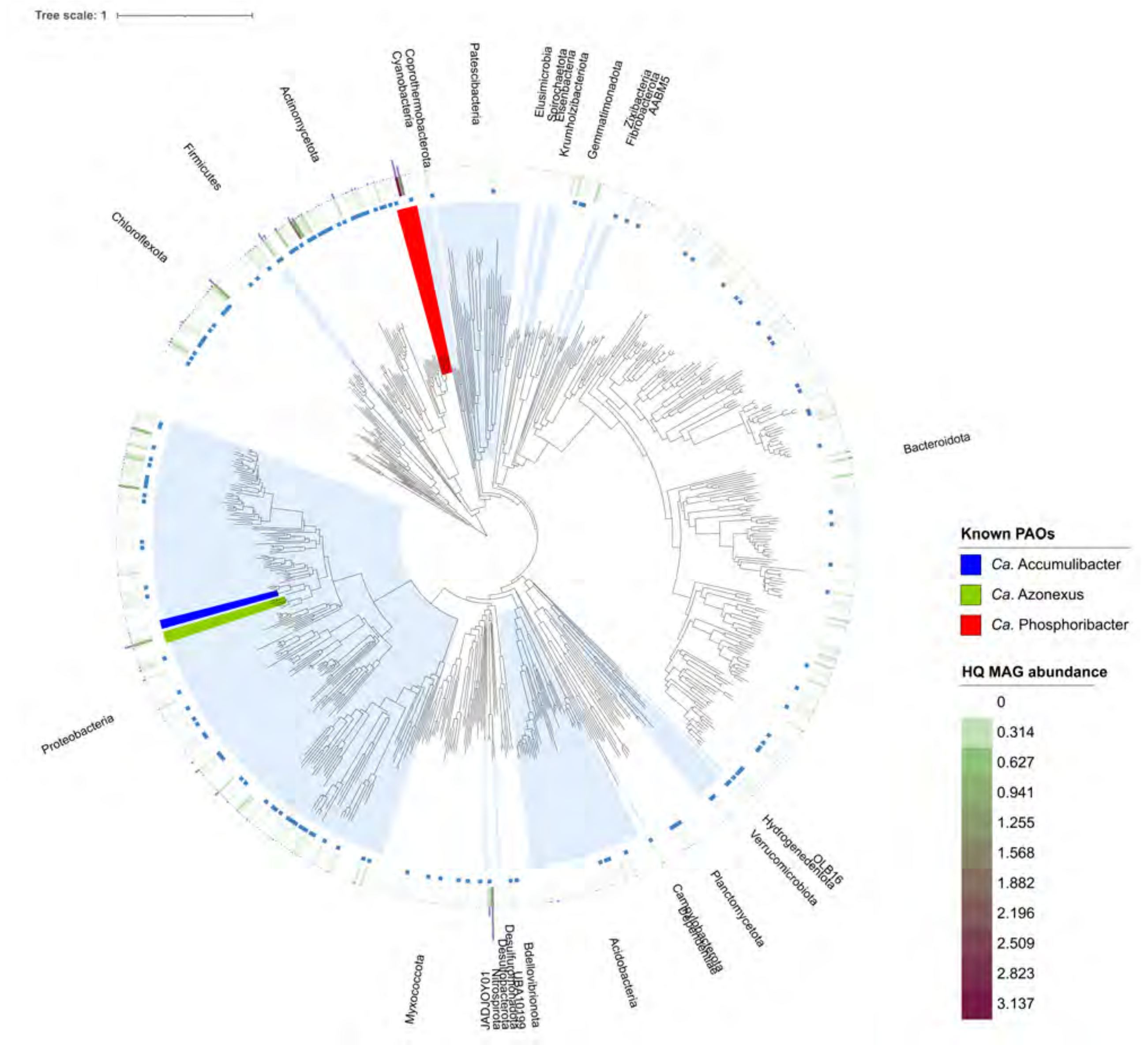

Figure S1. **Phylogenetic bacterial genome tree showing the diversity, abundance, expression, and recovered species.** The tree is based on the concatenated alignment of 120 single-copy marker gene proteins using GTDB-Tk v2.3.0. The 692 HQ bacterial species representatives are shown. MAGs representing PAOs are indicated by colors (green – *Ca.* Azonexus, blue – *Ca.* Accumulibacter, and red – *Ca*. Phosphoribacter). Light blue color indicates alternating phyla groups for visualization purposes. HQ-MAGs recovered in this study are indicated with the blue squares. The average relative abundance in the aeration tank of AAW WWTP of the MAG is indicated by the heatmap. Transcriptomic expression of each MAG is indicated by the purple bar chart. Additional information on the MAGs is present in **Supplementary data 2.**

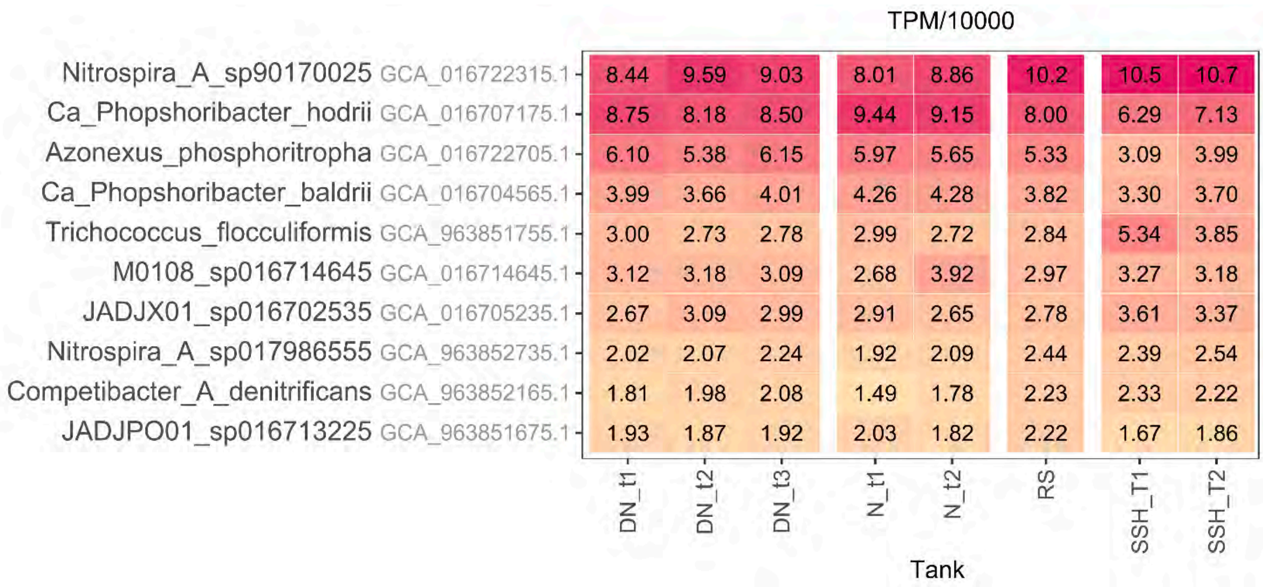

Figure S2. General expression of the species representatives in different WWTP tanks. Heatmap showing changes in expression levels within different AAW full-scale WWTP tanks of the top 10 MAGs. The bacterial taxonomy is based on GTDB-Tk. tX - represents different time points, T1/2 – represents different SSH tanks.

2.1 Metabolic prediction

2.1.1 Carbon degradation

All the MAGs had the genes for the uptake and degradation of amino acids and their storage. All of the PAOs showed potential for beta-oxidation, although genes for fatty acid transport (Black and DiRusso, 2003), were present only in *Ca.* Accumulibacter and *Ca.* Azonexus. In addition, based on Kofam koala annotation, *Ca.* Phosphoribacter species were missing genes for the EC1.1.1.35/1.1.1.211 reaction, but they were annotated with the Microscope platform.

2.1.2 Poly-P metabolism

We investigated two other gene sets related to phosphate assimilation, *phoRBPAD* (present in *Ca.* Accumulibacter and *Ca.* Azonexus), and *senX3, regX3, phoA,* and *pstS* (present in *Ca.* Phosphoribacter), which constitute a two-component system. Additionally, the *ppk1* and *ppk2* genes are involved in poly-P synthesis and degradation, and were found in all three PAO genera. In addition to *ppk2,* there are 6 other genes (*ppa, pap, ppaX, ppx, ppnk,* and *ppgk*) related to poly-P hydrolysis (Wang et al., 2018). All the genes are widespread in three PAO genera, with *ppnk* and *ppx* genes annotated in all the analyzed MAGs.

2.1.3 Storage polymers

There are two storage polymers that are associated with most PAOs: glycogen and PHA. Before *Ca.* Phosphoribacter (*Tetrasphaera*-like bacteria) was confirmed to be a PAO (Singleton et al., 2022), it was though that glycogen and PHA were essential for the PAO metabolism, however, based on metabolic reconstruction and *in situ* analysis, neither of these storage polymers were found in *Ca.* Phosphoribacter. It was discussed and proposed that *Ca.* Phosphoribacter could store different types of PHA storage polymers (PHV and not PHB) (Byrom, 1993), however, based on KofamKOALA

annotation, all of the MAGs from this genus lacked *phbB* genes, which however were annotation with the Microscope platform for all except *Ca.* P. baldri. So it is still uncertain whether PHA is an important storage polymer for this genus and it has not been detected using *in situ* Raman analyses. Cyanophycin is another storage polymer proposed to be present in PAOs, as the genes for cyanophycin synthesis and degradation were found in *Ca.* Phosphoribacter (Singleton et al., 2022; Zou et al., 2022). In *Ca.* Azonexus and *Ca.* Accumulibacter, only the *cphA* gene for cyanophycin synthesis were detected, so it is still uncertain whether this storage compound is important for PAOs.

2.1.4 Nitrogen cycle

Involvement in the nitrogen cycle is another metabolism of interest for the PAOs. So far, only members of *Ca.* Azonexus are considered full denitrifying PAO (DPAO) as some of the species can perform the entire denitrification process. Based on metabolic reconstruction, all species of the analyzed PAOs have the potential to be involved in different parts of the nitrogen cycle. However, only two reactions (NO_3^- reduction to NO_2^- and NO_2^- reduction to NO) were common for members from *Ca.* Accumulibacter, *Ca.* Azonexus, and *Ca.* Phosphoribacter.

Table S2. Genes potentially involved and analyzed in this study regarding PAOs metabolism related to poly-P accumulation.

KO	Gene ID	E.C	Annotation
**P transport**			
K03306	*pit*	NA	inorganic phosphate transporter
K07220	*pap*	NA	uncharacterized protein
K02040	*pstS*	NA	phosphate transport system substrate-binding protein
K02038	*pstA*	NA	phosphate transport system permease protein
K02036	*pstB*	NA	phosphate transport system ATP-binding protein
K02037	*pstC*	NA	phosphate transport system permease protein
**Poly-P synthesis and hydrolysis**			
K00937	*ppk1*	2.7.4.1	polyphosphate kinase
K22468	*ppk2*	2.7.4.34	polyphosphate kinase
K23753	*ppk2; pap*	2.7.4.33	AMP-polyphosphate phosphotransferase
K01507	*ppa*	3.6.1.1	inorganic pyrophosphatase
K06019	*ppaX*	3.6.1.1	pyrophosphatase
K01524	*ppx*	3.6.1.11	exopolyphosphatase
K03787	*surE*	3.1.3.5; 3.1.3.6	5'/3'-nucleotidase
K00886	*ppgk*	2.7.1.63	polyphosphate glucokinase
K00858	*ppnK*	2.7.1.23	NADK; NAD+ kinase
K00970	*pcnB*	2.7.7.19	poly(A) polymerase
**PHA accumulation**			
K00626	*atoB*	2.3.1.9	acetyl-CoA C-acetyltransferase
K00023	*phbB*	1.1.1.36	acetyl-CoA reductase
K03821	*phaC*	2.3.1.304	poly[(R)-3-hydroxyalkanoate] polymerase subunit PhaC
K22881	*phaE*	2.3.1.304	poly[(R)-3-hydroxyalkanoate] polymerase subunit PhaE
K00022	*HADH*	1.1.1.35	3-hydroxyacyl-CoA dehydrogenase
K07516	*fadN*	1.1.1.35	3-hydroxyacyl-CoA dehydrogenase

K01825	*fadB*	1.1.1.35; 4.2.1.17; 5.1.2.3; 5.3.3.8	3-hydroxyacyl-CoA dehydrogenase
K01782	*fadJ*	1.1.1.35; 4.2.1.17; 5.1.2.3	3-hydroxyacyl-CoA dehydrogenase
K07514	*EHHADH*	1.1.1.35; 4.2.1.17; 5.3.3.8	3-hydroxyacyl-CoA dehydrogenase
K00074	*paaH*	1.1.1.157	3-hydroxyacyl-CoA dehydrogenase
K01692	*paaF*	4.2.1.17	enoyl-CoA hydratase
K07515	*HADHA*	4.2.1.17; 1.1.1.211	enoyl-CoA hydratase
K07511	*ECHS1*	4.2.1.17	enoyl-CoA hydratase
K01715	*crt*	4.2.1.17	enoyl-CoA hydratase
K17865	*croR*	4.2.1.55	3-hydroxybutyryl-CoA dehydrogenase
**Glycogen synthesis**			
K01835	*pgm*	5.4.2.2	phosphoglucomutase
K15779	*PGM2*	5.4.2.2; 5.4.2.7	phosphoglucomutase
K15778	*pmm-pgm*	5.4.2.8; 5.4.2.2	phosphoglucomutase
K00975	*glgC*	2.7.7.27	glucose-1-1phosphate adenylytransferase
K00703	*glgA*	2.4.1.21	starch synthase
K20812	*glgA*	2.4.1.242	glycogen synthase
K00700	*glgB*	2.4.1.18	1,4-alpha-glucan branching enzyme
K00963	*galU*	2.7.7.9	UTP-glucose-1-phosphate uridylytransferase
K00693	*GYS*	2.4.1.11	glycogen synthase
K00750	*GYG1*	2.4.1.186	glycogenic
K16150	*K16150*	2.4.1.11	glycogen synthase
K16153	*K16153*	2.4.1.1.1; 2.4.1.11	glycogen phosphorylase/synthase
**Glycogen degradation**			
K00688	*glgP*	4.2.1.1	glycogen phosphorylase
K16153	*K16153*	4.2.1.1; 2.4.1.11	glycogen phosphorylase
K01084	*G6PC*	3.1.3.9	glucose-6-phosphatase
K01214	*treX*	3.2.1.6	isomylase
K01176	*amy*	3.2.1.1	alpha-amylase
K07405	*E3.2.1.1A*	3.2.1.1	
K22451	*jgt*	2.4.1.25	4-alpha-glucantransferase
K00705	*malQ*	2.4.1.25	4-alpha-glucantransferase
K02438	*glgX*	3.2.1.196	glycogen debranching enzyme
K01200	*pulA*	3.2.1.41	
K05343	*treS*	3.2.1.1; 5.4.99.16	alpha-amylase
K21574	*susB*	3.2.1.3	glucan-1,4-alpha-glucosidase
K01178	*SGA1*	3.2.1.3	glucoamylase
K12047	*MGAM*	3.2.1.20; 3.2.1.3	maltase-glucoamylase
K01187	*malZ*	3.2.1.20	alpha-glucosidase
**Cyanophycin**			
K03803	*cphA*	6.3.2.29; 6.3.2.30	cyanophycin synthetase
K13282	*cphB*	3.4.15.6	cyanophycinase
**Nitrogen cycle**			
K00370	*narG*	1.7.5.1	nitrate reductase/nitrite oxidoreductase, alpha subunit
K00371	*narH*	1.7.5.1	nitrate reductase/nitrite oxidoreductase, beta subunit

K00374	*narI*	1.7.5.1	nitrate reductase gamma subunit
K02567	*napA*	1.9.6.1	nitrate reductase (cytochrome)
K02568	*napB*		nitrate reductase (cytochrome), electron transfer subunit
K00363	*nirD*	1.7.1.15	nitrite reductase (NADH) small subunit
K00362	*nirB*	1.7.1.15	nitrite reductase (NADH) large subunit
K03385	*nrfA*	1.7.2.2	nitrite reductase (cytochrome c-552)
K15876	*nrfH*		cytochrome c nitrite reductase small subunit
K00367	*narB*	1.7.7.2	ferredoxin-nitrate reductase
K10534	*NR*	1.7.1.1; 1.7.1.2; 1.7.1.3	nitrate reductase (NAD(P)H)
K00372	*nasA*	1.7.99.-	assimilatory nitrate reductase catalytic subunit
K00360	*nasB*	1.7.99.-	assimilatory nitrate reductase electron transfer subunit
K17877	*NIT-6*	1.7.1.4	nitrate reductase (NAD(P)H)
K00366	*nirA*	1.7.7.1	ferredoxin-nitrite reductase
K00361	*nasB*	1.7.1.4	nitrite reductase [NAD(P)H]
K26139	*nasD*	1.7.1.4	nitrite reductase [NAD(P)H] large subunit
K26138	*nasE*	1.7.1.4	nitrite reductase [NAD(P)H] small subunit
K00368	*nirK*	1.7.2.1	nitrite reductase (NO-forming)
K15864	*nirS*	1.7.2.1; 1.7.99.1	nitrite reductase (NO-forming) / hydroxylamine reductase
K04561	*norB*	1.7.2.5	nitric oxide reductase subunit B
K02305	*norC*		nitric oxide reductase subunit C
K00376	*nosZ*	1.7.2.4	nitrous-oxide reductase
K02586	*nifD*	1.18.6.1	nitrogenase molybdenum-iron protein alpha chain
K02591	*nifK*	1.18.6.1	nitrogenase molybdenum-iron protein beta chain
K02588	*nifH*		nitrogenase iron protein NifH
K00531	*anfG*	1.18.6.1	nitrogenase delta subunit
K22896	*vnfD*	1.18.6.2	vanadium-dependent nitrogenase alpha chain
K22897	*vnfK*	1.18.6.2	vanadium-dependent nitrogenase beta chain
K22898	*vnfG*	1.18.6.2	vanadium nitrogenase delta subunit
K22899	*vnfH*		vanadium nitrogenase iron protein
K10535	*Hoa*	1.7.2.6	hydroxylamine dehydrogenase
K10946	*amoC*		methane/ammonia monooxygenase subunit C
K10944	*amoA*	1.14.18.3; 1.14.99.39	methane/ammonia monooxygenase subunit A
K10945	*amoB*		methane/ammonia monooxygenase subunit B
K20932	*Hzs*	1.7.2.7	hydrazine synthase subunit
K20933	*Hzs*	1.7.2.7	hydrazine synthase subunit
K20934	*Hzs*	1.7.2.7	hydrazine synthase subunit
K20935	*Hdh*	1.7.2.8	hydrazine dehydrogenase

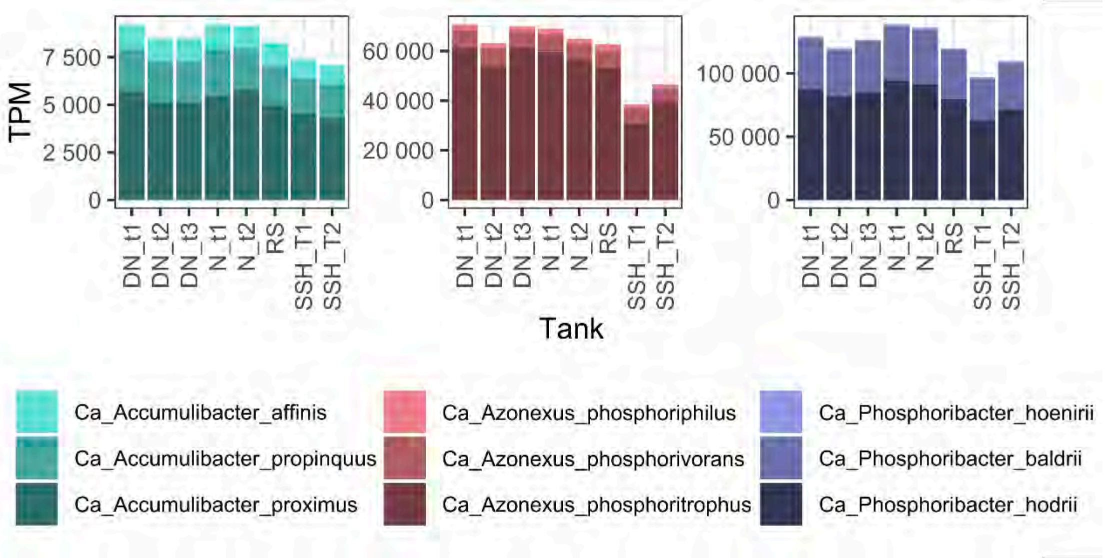

Figure S3. General activity of PAO species in different tanks of a full-scale WWTP. The graphs show total expression levels of the PAO species over the time course of anoxic incubations with different C-sources.

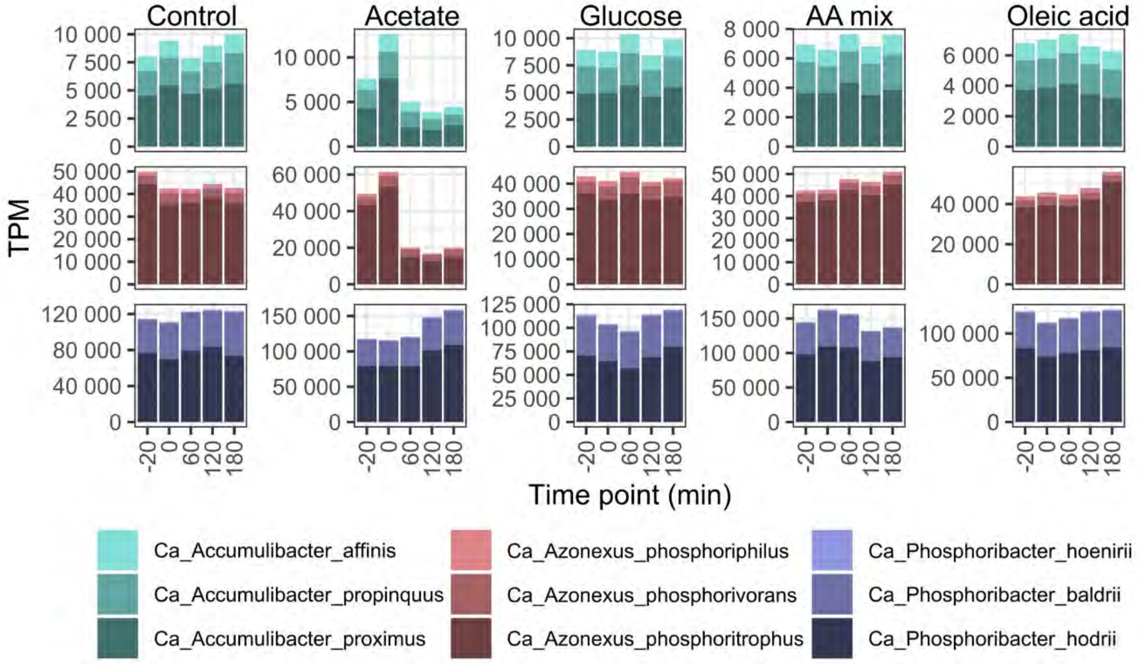

Figure S4. General activity of PAO species with different carbon sources. The graphs show total expression levels of the PAO species over the time course of anoxic incubations with different C-sources.

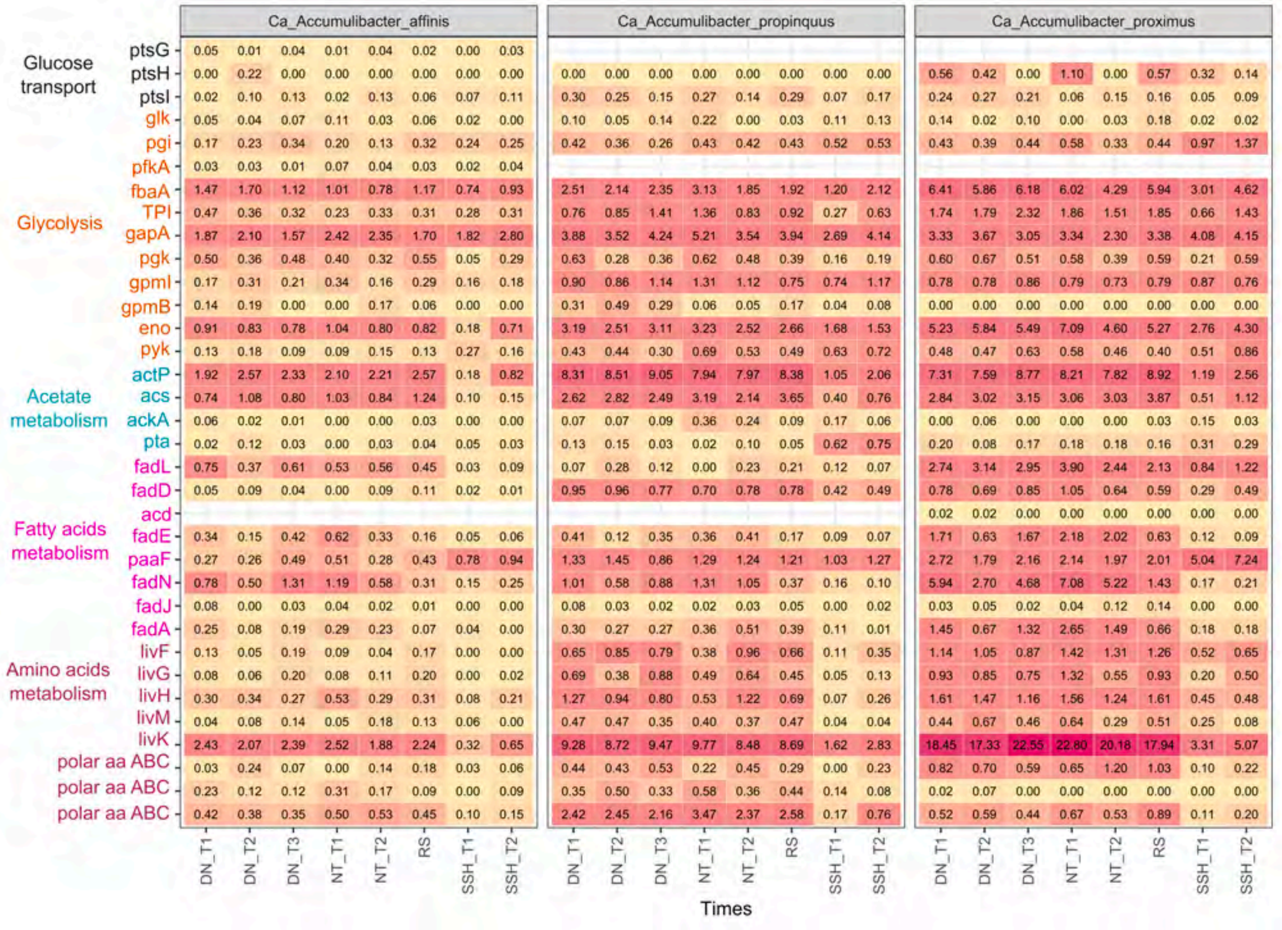

Figure S5. Expression of the genes related to the carbon source in *Ca.* Accumulibacter. Heatmap shows expression of the genes in relation to carbon source metabolism of three *Ca.* Accumulibacter species in different tanks of full-scale WWTP.

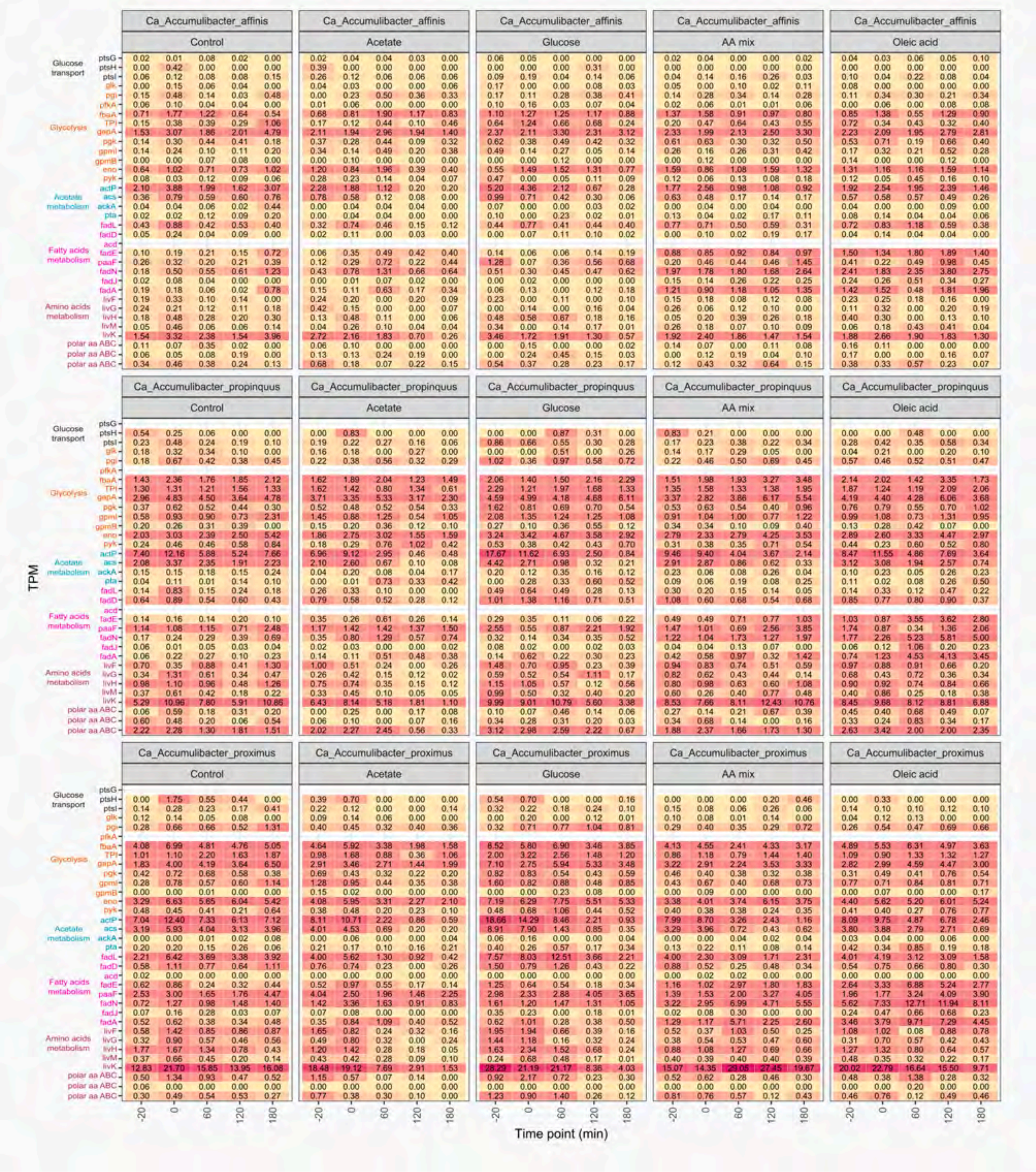

Figure S6. Expression of the genes related to the carbon source in *Ca.* Accumulibacter with addition of different carbon sources. Heatmap shows expression of the genes in relation to carbon source metabolism of three *Ca.* Accumulibacter species in incubation experiments, under anoxic conditions with different carbon sources.

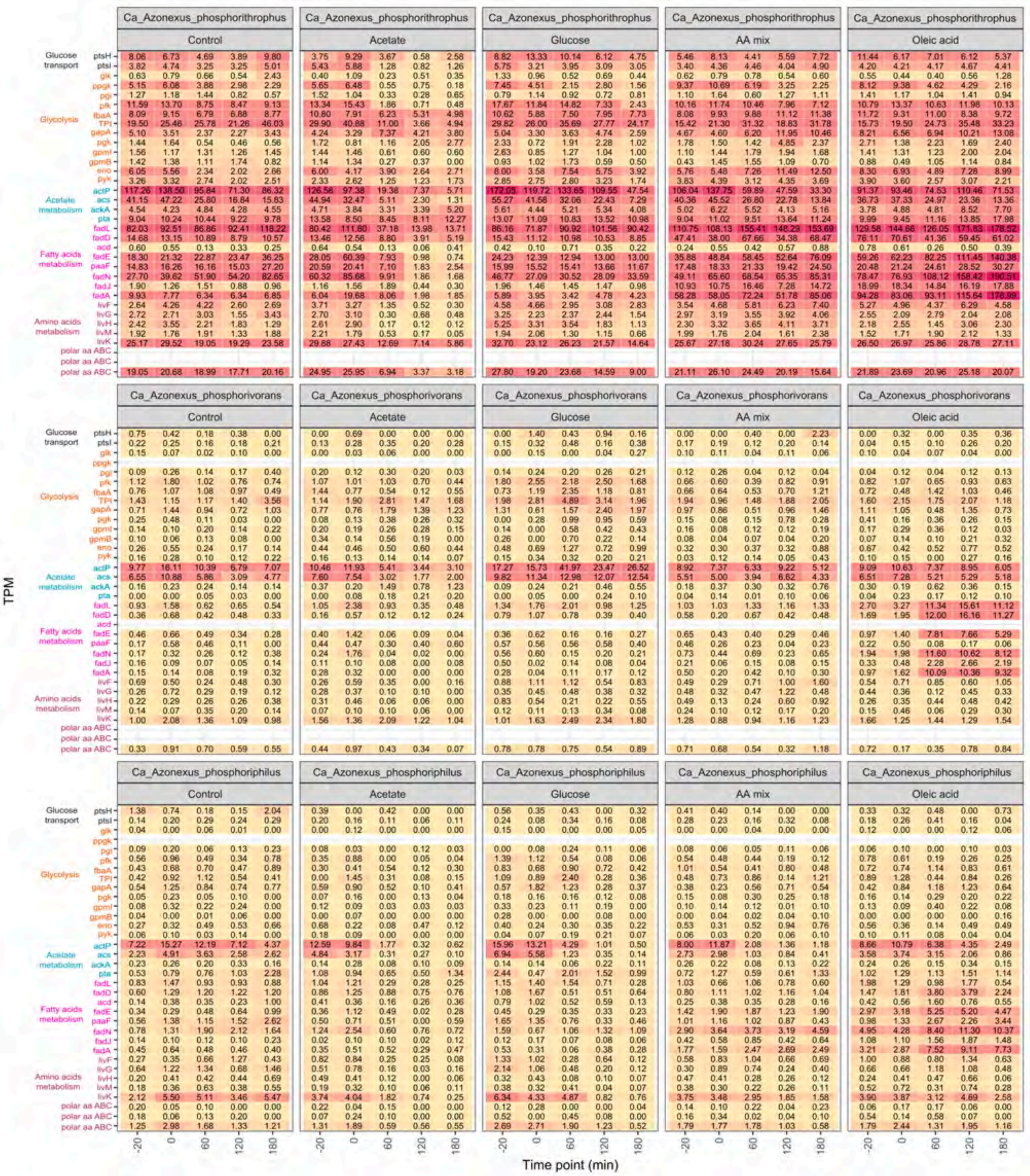

Figure S7. Expression of the genes related to the carbon source in *Ca.* Azonexus with addition of different carbon sources. Heatmap shows expression of the genes in relation to carbon source metabolism of three *Ca.* Azonexus species in incubation experiments under anoxic conditions with different carbon sources.

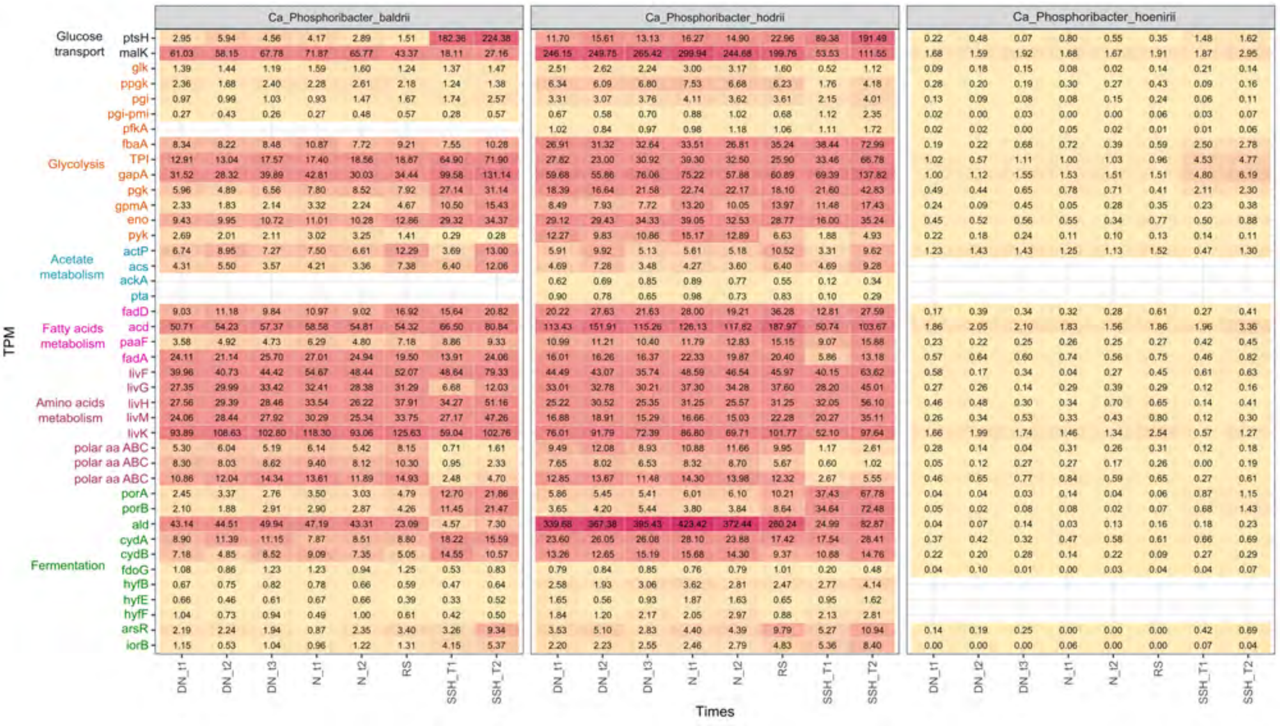

Figure S8. Expression of the genes related to the carbon source in *Ca.* Phosphoribacter in a full-scale WWTP. Heatmap shows expression of the genes in relation to carbon source metabolism of three *Ca.* Phosphoribacter species in different tanks of full-scale WWTP.

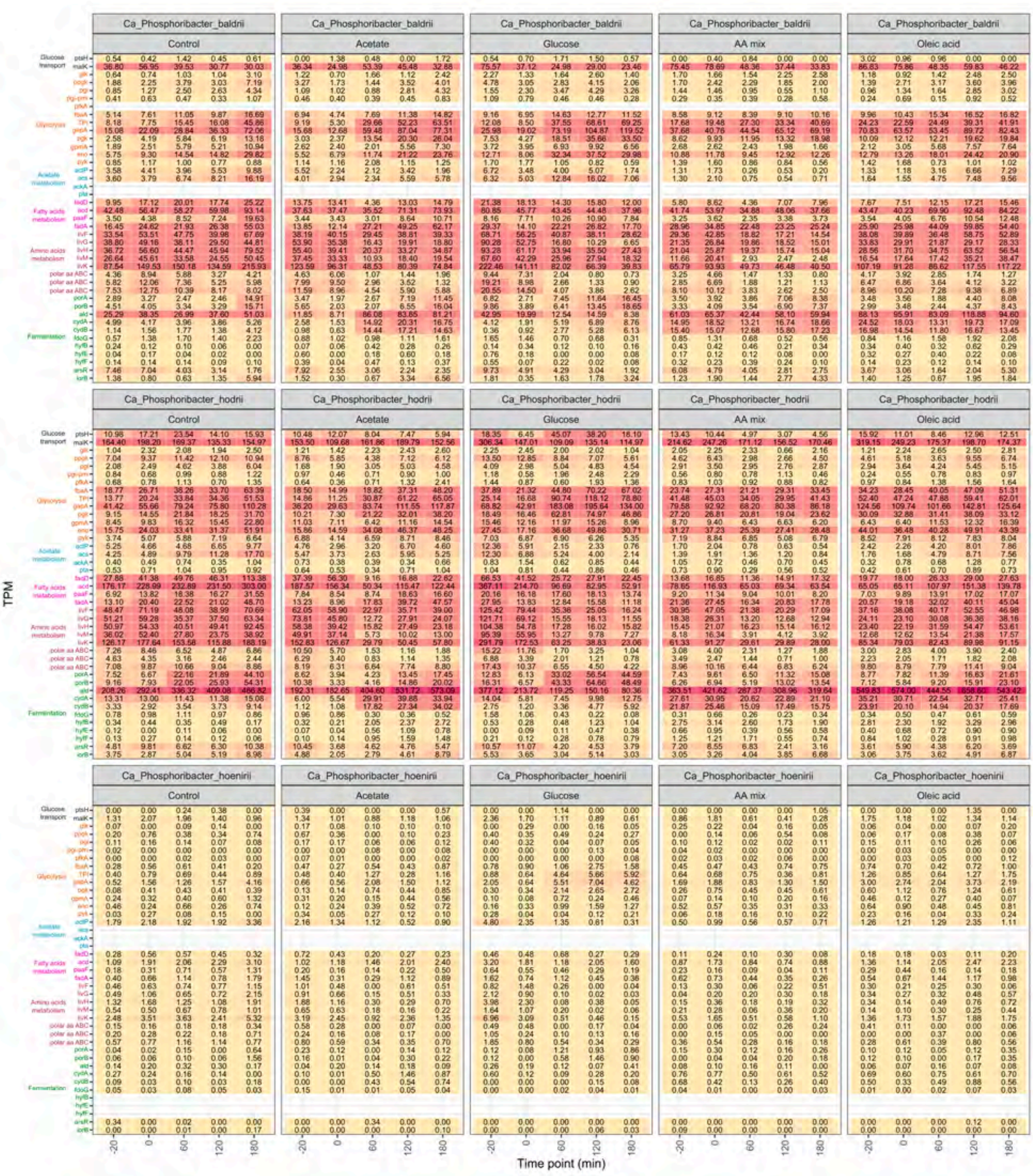

Figure S9. Expression of the genes related to the carbon source in *Ca.* Phosphoribacter with addition of different carbon sources. Heatmap shows expression of the genes in relation to carbon source metabolism of three *Ca.* Phosphoribacter species in incubation experiments under anoxic conditions with different carbon sources.

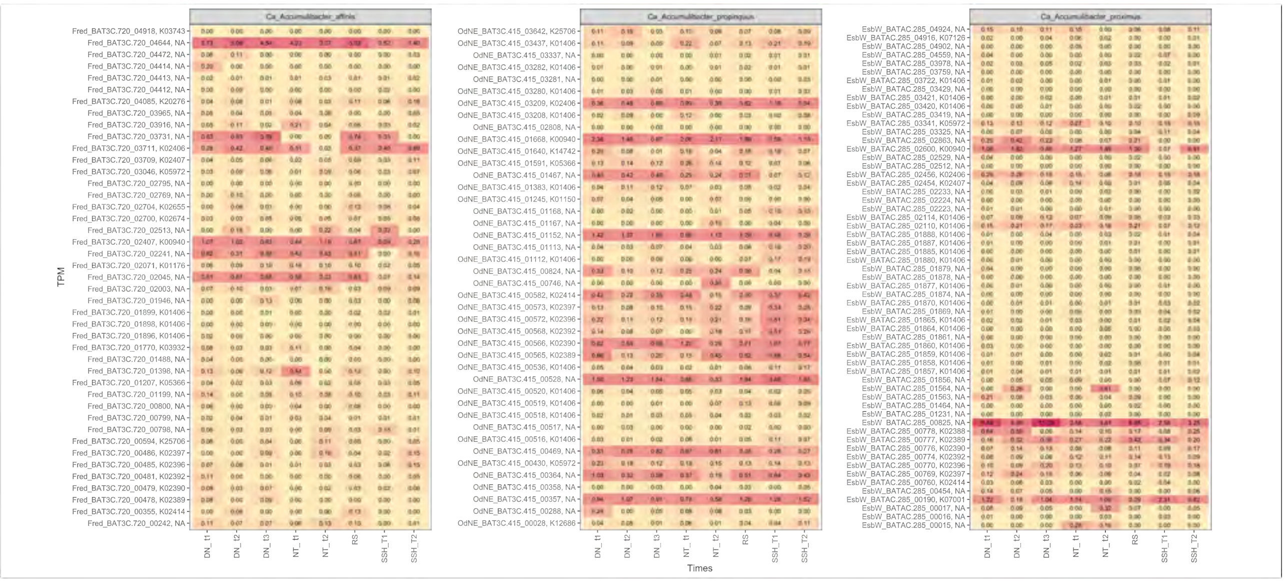

Figure S10. Expression of the genes encoding extracellular enzymes in *Ca.* Accumulibacter in different tanks of full-scale WWTP.

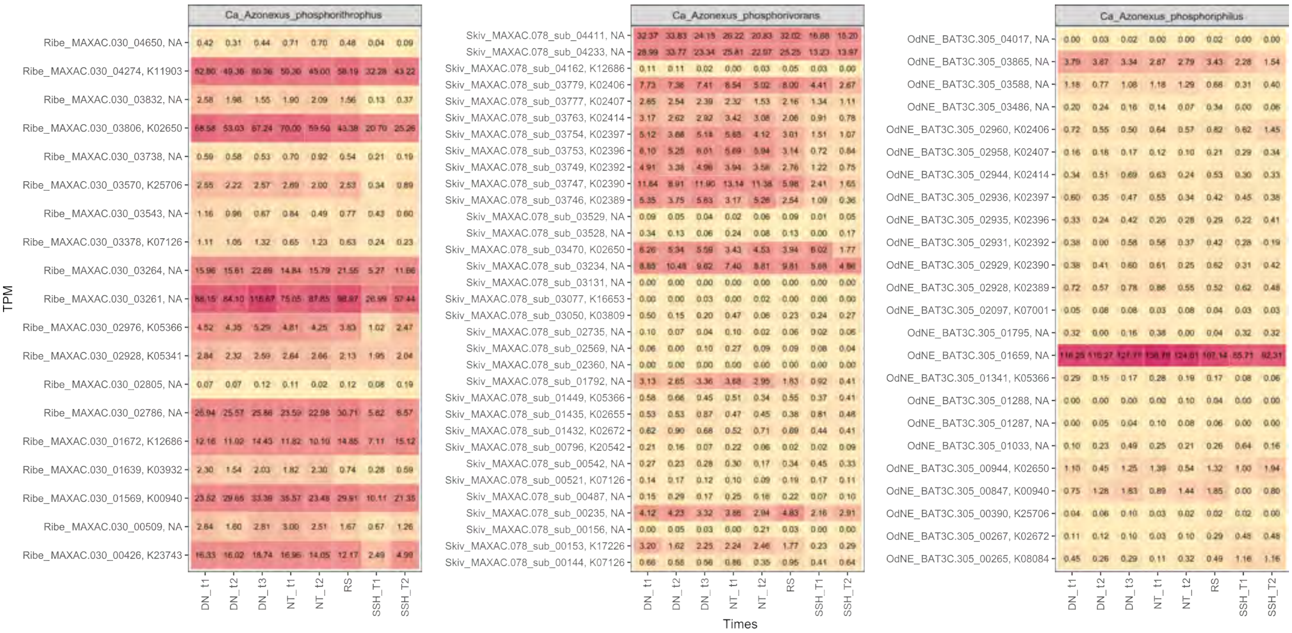

Figure S11. Expression of the genes encoding extracellular enzymes in *Ca.* Azonexus in different tanks of full-scale WWTP.

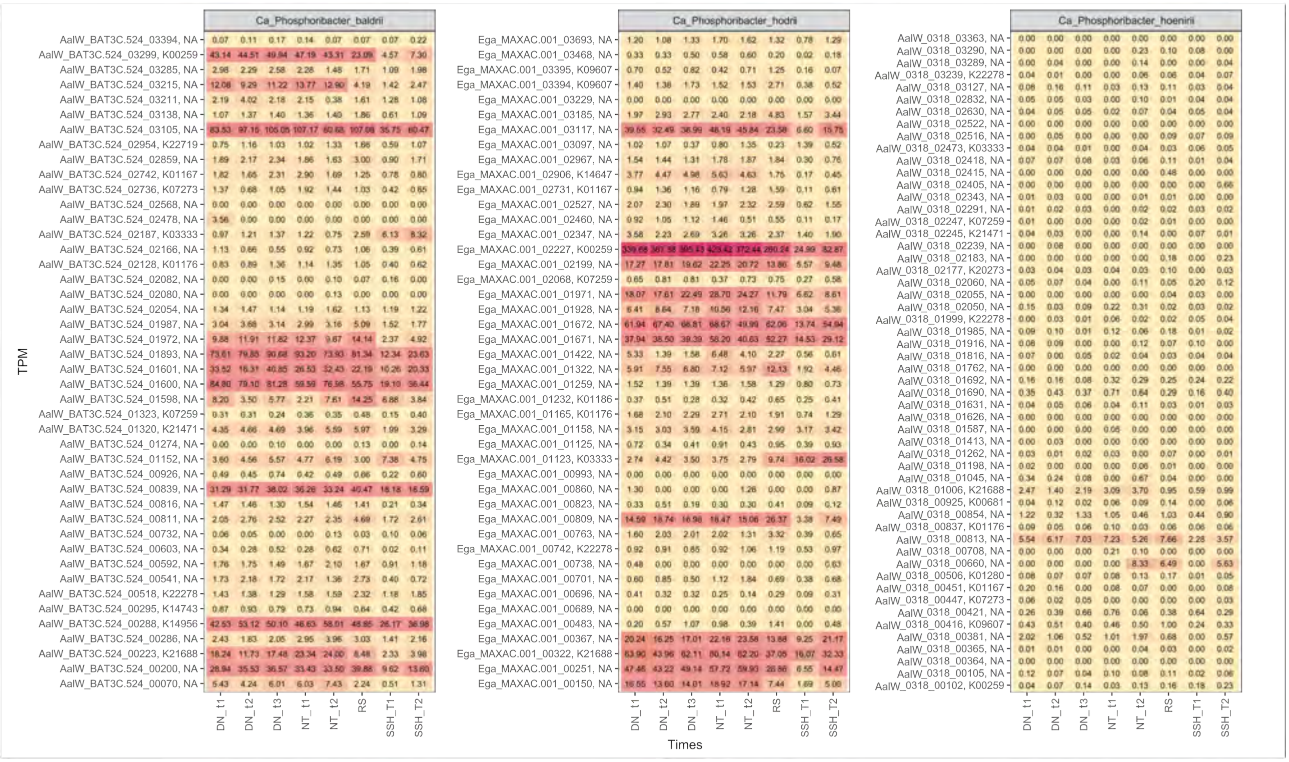

Figure S12. Expression of the genes encoding extracellular enzymes in *Ca.* Phosphoribacter in different tanks of full-scale WWTPs.

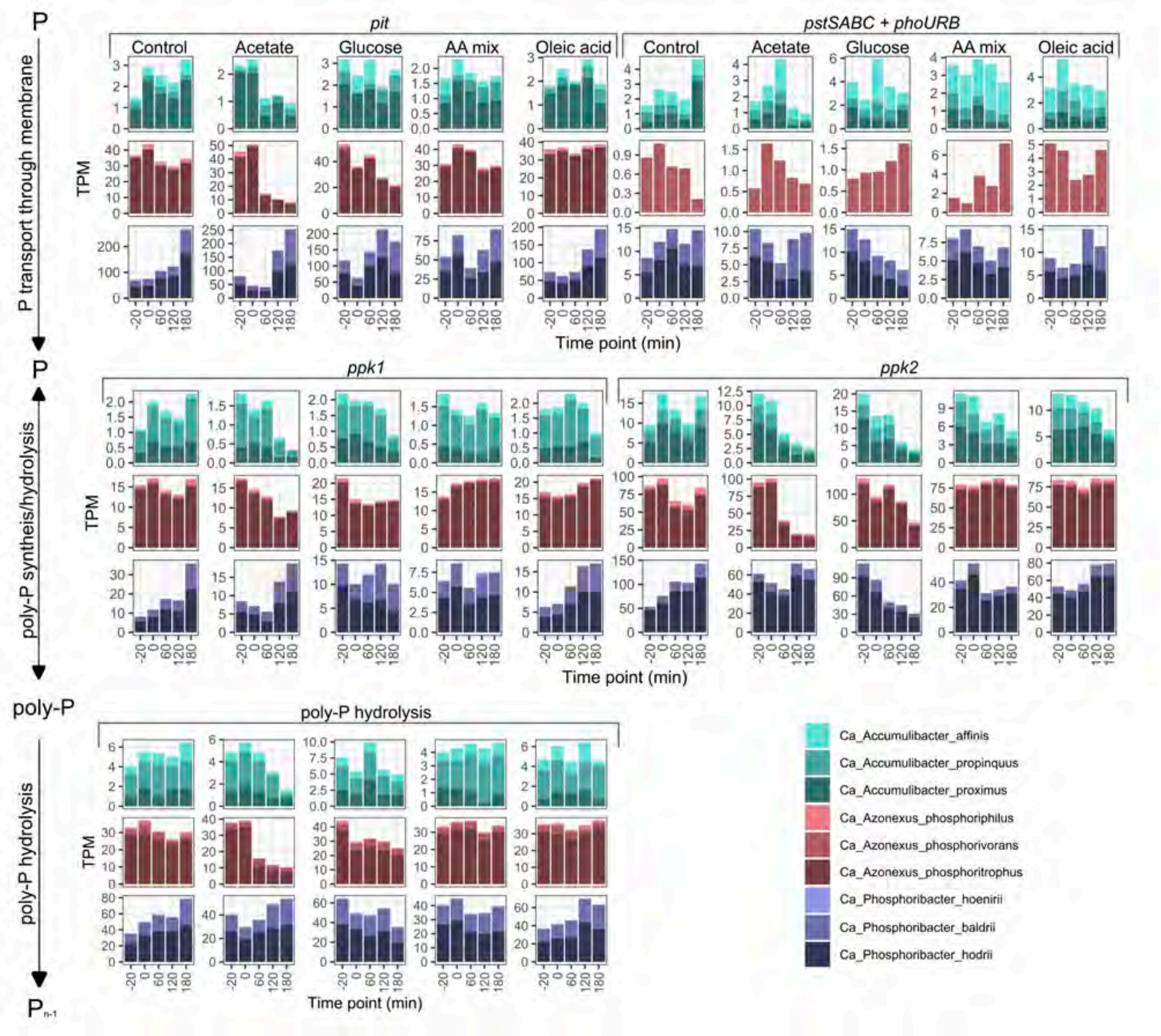

Figure S13. Expression levels of genes related to poly-P metabolism in short-term incubation with different C sources. The graphs show changes in expression levels of genes related to P transport, synthesis, and hydrolysis in short-term incubations with different C-sources.

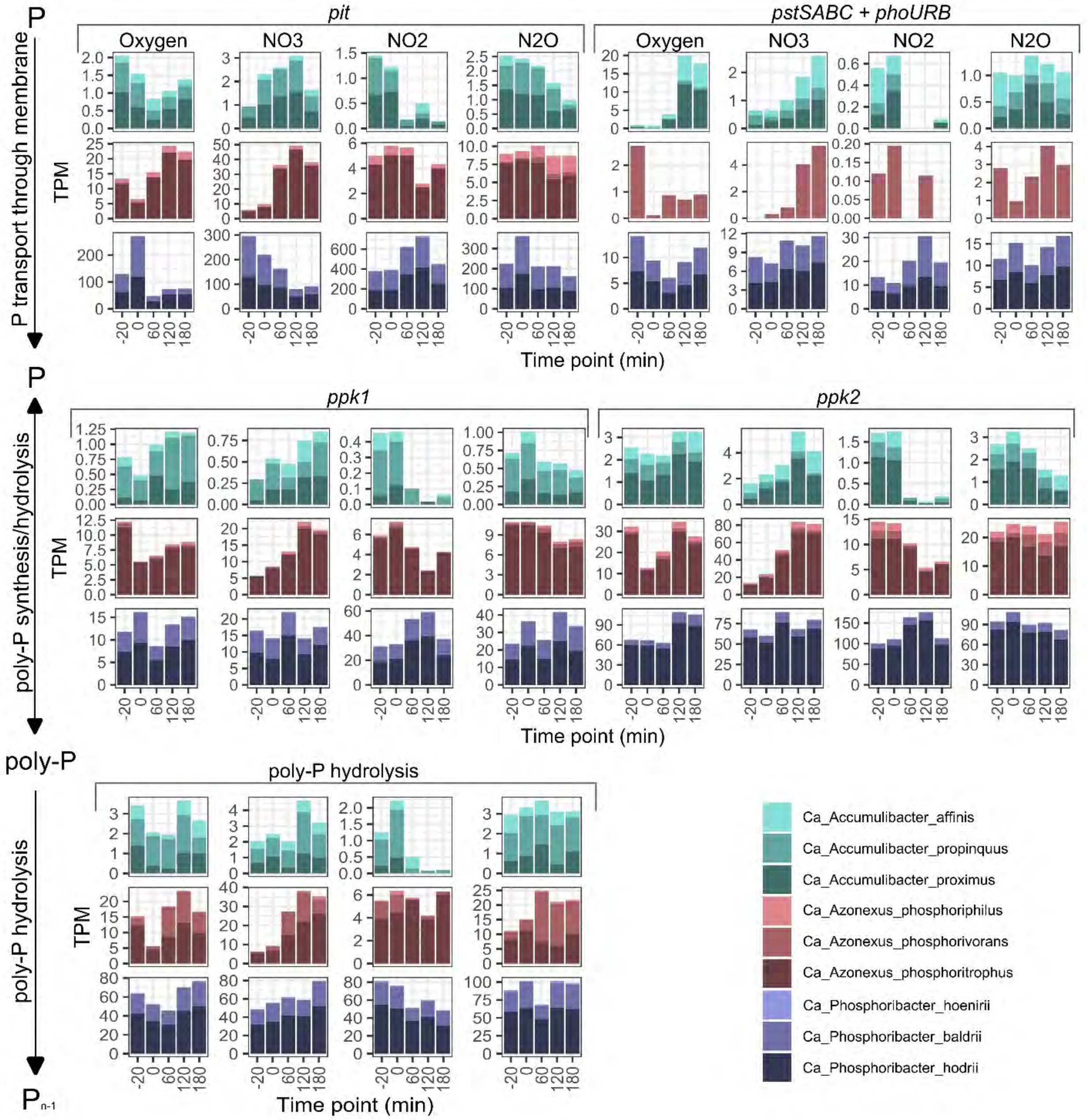

Figure S14. Expression levels of genes related to poly-P metabolism in short-term incubation with different electron acceptors. The graphs show changes in expression levels of genes related to P transport, synthesis, and hydrolysis in short-term incubations with different electron acceptors.

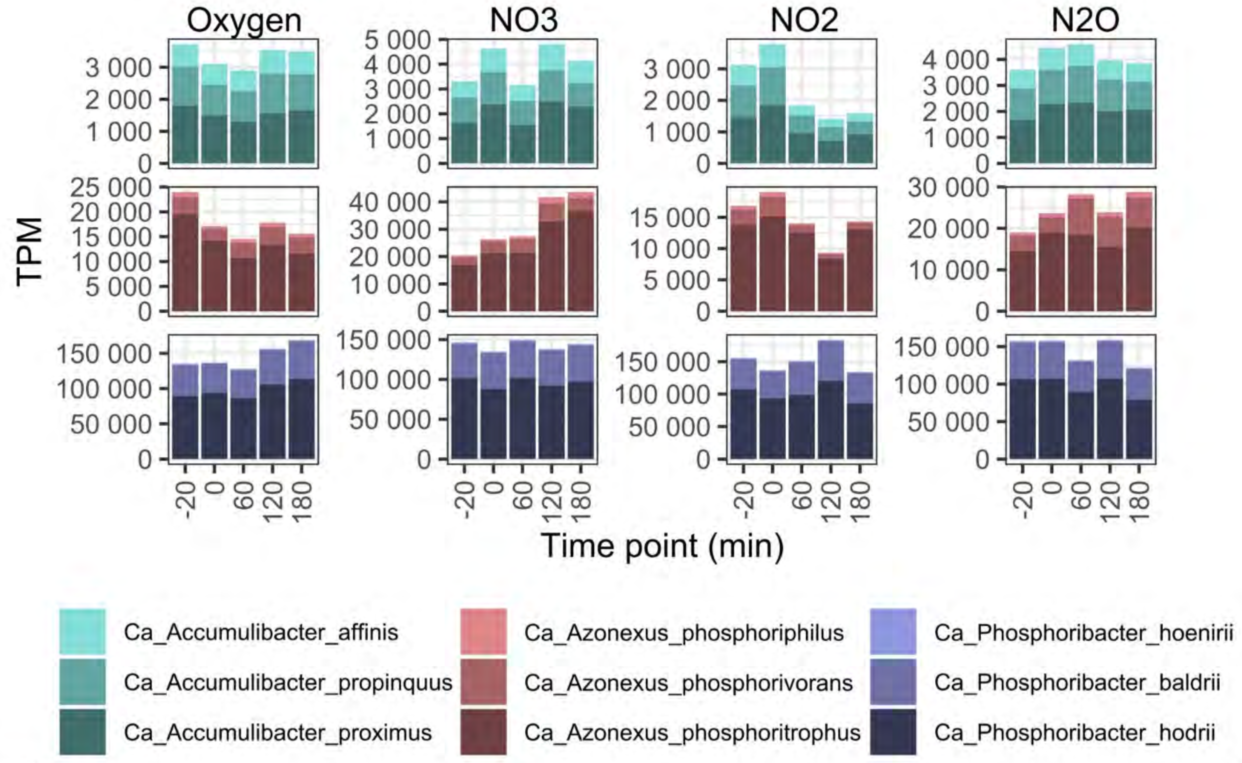

Figure S15. General activity of the three PAOs genera. The graphs show total expression levels of the PAO species over the time of short-term incubations under the influence of different electron acceptors.

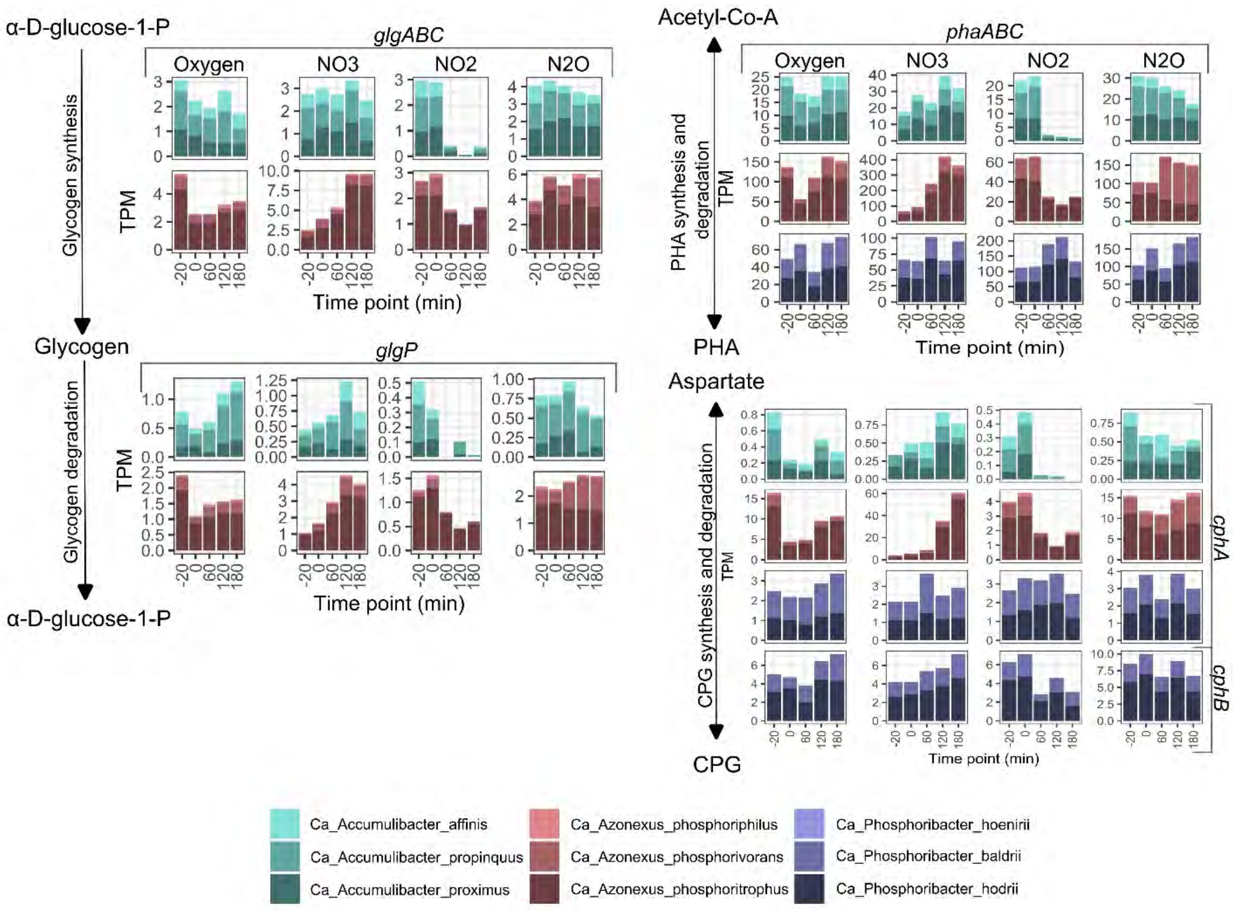

Figure S16. Expression levels of genes related to storage polymer metabolism in short-term incubations with different electron acceptors. The graphs show changes in expression levels of genes related to glycogen, PHA, and cyanophycin (CPG) metabolism in short-term incubations with different electron acceptors.

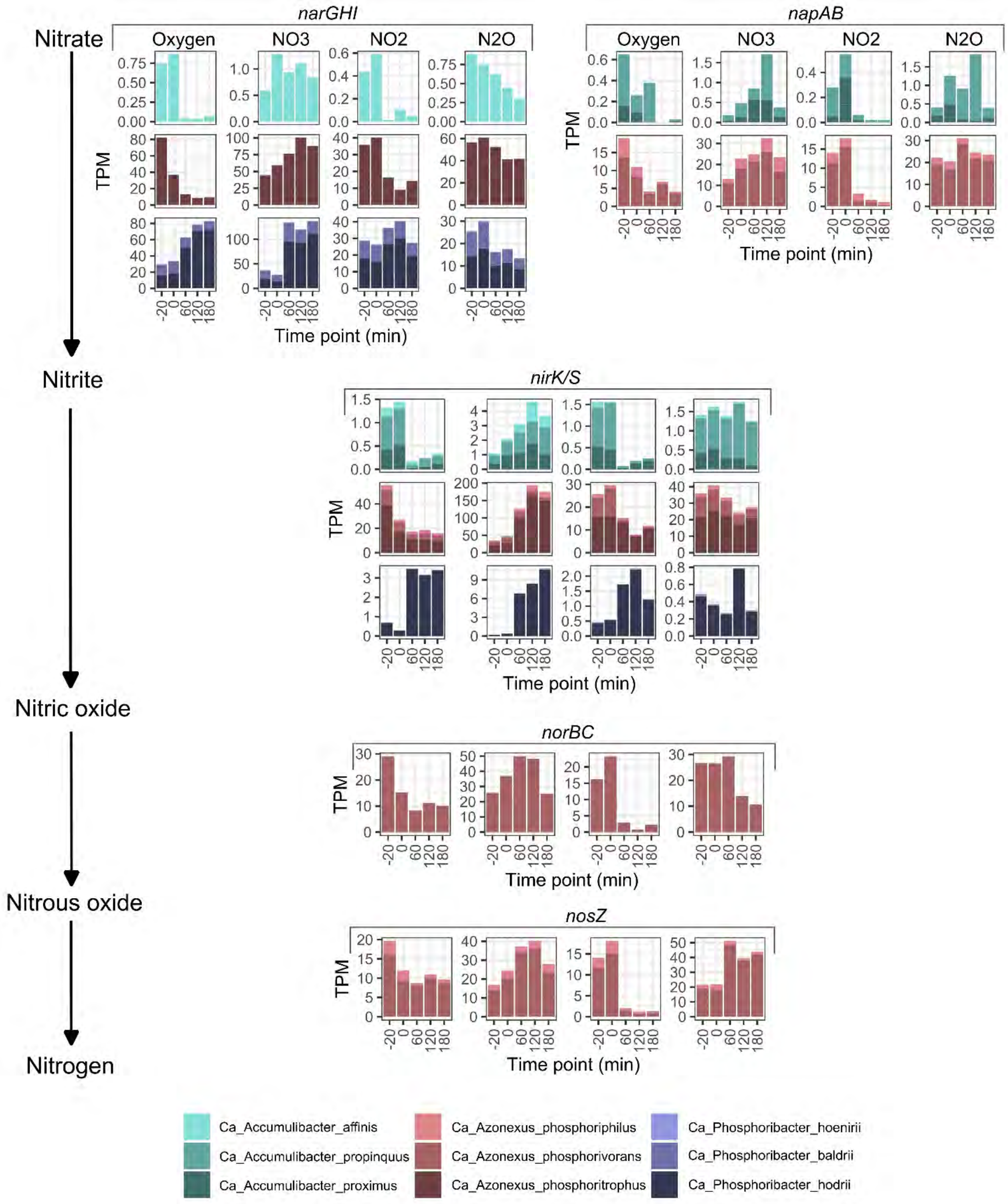

Figure S17. Expression levels of genes related to the denitrification process. The graph shows changes in expression levels of genes related to the denitrification process in PAO species during short-term incubations with different electron acceptors.

3 Taxonomic proposal

Table S3. Protologues table for *Ca.* Phosphoribacter tyrii.

Species name	*Candidatus* Phosphoribacter tyrii
Genus name	*Candidatus* Phosphoribacter
Specific epithet	tyrii
Type species of the genus	*Candidatus* Phosphoribacter baldrii
Genus status	Candidatus
Species etymology	"*Candidatus* Phosphoribacter tyrii" sp. nov.: ty'ri.i. N.L. gen. n. *tyrii* of Tyr, Norse god of war and justice.
Species status	sp. nov.
Designation of the type MAG	GCA_016711095.1
MAG/SAG accession number	GCA_016711095.1
Genome status	High-quality draft
Genome size	2919710
GC mol %	68.92
Country of origin	Denmark
Region of origin	Hirtshals
Source of sample	Full-scale enriched biological phosphorus removal wastewater treatment plant
Geographical location	Hirtshals
Latitude	57.577275
Longitude	9.992971
Depth	N/A
Altitude	N/A
Temperature of the sample	Mesophilic
pH of the sample	N/A
Relationship to oxygen	facultative anaerobe
Energy metabolism	Likely utilizes a range of substrates including sugars and amino acids, and is capable of polyphosphate accumulation
Assembly	1 sample
Sequencing technology	Oxford Nanopore and Illumina Hiseq X
Binning software used	MetaBAT2
Assembly software used	CANU v1.8
Habitat	Full-scale biological phosphorus removal wastewater treatment plant
Miscellaneous, extraordinary features relevant for the description	N/A

Table S4. Protologues table for *Ca.* Phosphoribacter thorii.

Species name	*Candidatus* Phosphoribacter thorii
Genus name	*Candidatus* Phosphoribacter
Specific epithet	thorii
Type species of the genus	*Candidatus* Phosphoribacter baldrii
Genus status	Candidatus
Species etymology	*"Candidatus* Phosphoribacter thorii" sp. nov.: tho´ri.i. N.L. gen. n. *thorii* of Thor, Norse god of thunder.
Species status	sp. nov.
Designation of the type MAG	GCA_016715285.1
MAG/SAG accession number	GCA_016715285.1
Genome status	High-quality draft
Genome size	3282635
GC mol %	66.66
Country of origin	Denmark
Region of origin	Mariagerfjord
Source of sample	Full-scale enriched biological phosphorus removal wastewater treatment plant
Geographical location	Mariagerfjord
Latitude	56.742252
Longitude	10.114475
Depth	N/A
Altitude	N/A
Temperature of the sample	Mesophilic
pH of the sample	N/A
Relationship to oxygen	facultative anaerobe
Energy metabolism	Likely utilizes a range of substrates including sugars and amino acids, and is capable of polyphosphate accumulation
Assembly	1 sample
Sequencing technology	Oxford Nanopore and Illumina Hiseq X
Binning software used	MetaBAT2
Assembly software used	CANU v1.8
Habitat	Full-scale biological phosphorus removal wastewater treatment plant
Miscellaneous, extraordinary features relevant for the description	N/A

Table S5. Protologues table for *Ca.* Phosphoribacter freyrii.

Species name	*Candidatus* Phosphoribacter freyrii
Genus name	*Candidatus* Phosphoribacter
Specific epithet	freyrii
Type species of the genus	*Candidatus* Phosphoribacter baldrii
Genus status	Candidatus
Species etymology	*"Candidatus* Phosphoribacter freyrii" sp. nov.: fre´y.ri.i. N.L. gen. n. freyrii of Freyr, Norse god of fertility.
Species status	sp. nov.
Designation of the type MAG	GCA_016710385.1
MAG/SAG accession number	GCA_016710385.1
Genome status	High-quality draft
Genome size	3828964
GC mol %	69.12
Country of origin	Denmark
Region of origin	Hjorring
Source of sample	Full-scale enriched biological phosphorus removal wastewater treatment plant
Geographical location	Hjorring
Latitude	57.421265
Longitude	9.975411
Depth	N/A
Altitude	N/A
Temperature of the sample	Mesophilic
pH of the sample	N/A
Relationship to oxygen	Facultative anaerobe
Energy metabolism	Likely utilizes a range of substrates including sugars and amino acids, and is capable of polyphosphate accumulation
Assembly	1 sample
Sequencing technology	Oxford Nanopore and Illumina Hiseq X
Binning software used	MetaBAT2
Assembly software used	CANU v1.8
Habitat	Full-scale biological phosphorus removal wastewater treatment plant
Miscellaneous, extraordinary features relevant for the description	N/A

Table S6. Protologues table for *Ca.* Phosphoribacter hoenirii.

Species name	*Candidatus* Phosphoribacter hoenirii
Genus name	*Candidatus* Phosphoribacter
Specific epithet	hoenirii
Type species of the genus	*Candidatus* Phosphoribacter baldrii
Genus status	Candidatus
Species etymology	Description of *"Candidatus* Phosphoribacter hoenirii" sp. nov.: ho´e.ri.i. N.L. gen. n. *hoenirii* of Hoenir, Norse god of silence.
Species status	sp. nov.
Designation of the type MAG	GCA_963851545.1
MAG/SAG accession number	GCA_963851545.1
Genome status	High-quality draft
Genome size	3524743
GC mol %	69.13
Country of origin	Denmark
Region of origin	Aalborg West
Source of sample	Full-scale enriched biological phosphorus removal wastewater treatment plant
Geographical location	Aalborg West
Latitude	57.0480347
Longitude	9.8654226
Depth	N/A
Altitude	N/A
Temperature of the sample	Mesophilic
pH of the sample	N/A
Relationship to oxygen	Facultative anaerobe
Energy metabolism	Likely utilizes a range of substrates including sugars and amino acids, and is capable of polyphosphate accumulation
Assembly	1 sample
Sequencing technology	Oxford Nanopore and Illumina Hiseq X
Binning software used	MetaBAT2
Assembly software used	CANU v1.8
Habitat	Full-scale enhanced biological phosphorus removal wastewater treatment plant
Miscellaneous, extraordinary features relevant for the description	N/A

Table S7. Protologues table for *Ca.* Azonexus phosphoriphilus

Species name	*Candidatus* Azonexus phosphoriphilus
Genus name	*Candidatus* Azonexus
Specific epithet	phosphoriphilus
Type species of the genus	*Azonexus agitata* strain CKB
Genus status	Validly published
Species etymology	Description of *"Candidatus* Azonexus phosphoriphilus" sp. nov.: phos.pho.ri.phi´lus. N.L. masc. n. *phosphorus*, phosphorus; G. masc. n. *philos*, lover; N.L. masc. adj. *phosphoriphilus*, indicating the ability to uptake phosphorus.
Species status	sp. nov.
Designation of the type MAG	GCA_016714975.1
MAG/SAG accession number	GCA_016714975.1
Genome status	High-quality draft
Genome size	4076926
GC mol %	62.08
Country of origin	Denmark
Region of origin	Odense NE
Source of sample	Full-scale enriched biological phosphorus removal wastewater treatment plant
Geographical location	Odense NE
Latitude	55.432604
Longitude	10.458855
Depth	N/A
Altitude	N/A
Temperature of the sample	Mesophilic
pH of the sample	N/A
Relationship to oxygen	Facultative anaerobe
Energy metabolism	Potentially utilizing acetate and amino acids
Assembly	1 sample
Sequencing technology	Oxford Nanopore and Illumina Hiseq X
Binning software used	MetaBAT2
Assembly software used	CANU v1.8
Habitat	Full-scale enhanced biological phosphorus removal wastewater treatment plant
Miscellaneous, extraordinary features relevant for the description	N/A

Table S8. Protologues table for *Ca.* Azonexus defluvii.

Species name	*Candidatus* Azonexus defluvii
Genus name	*Candidatus* Azonexus
Specific epithet	defluvii
Type species of the genus	*Azonexus agitata* strain CKB
Genus status	Validly published
Species etymology	Description of *"Candidatus* Azonexus defluvii" sp. nov.: de.flu´vi.i. L. masc. gen. n. *defluvii*, of sewage. This taxon is represented by the MAG GCA_016709495
Species status	sp. nov.
Designation of the type MAG	GCA_016709495.1
MAG/SAG accession number	GCA_016709495.1
Genome status	High-quality draft
Genome size	4450621
GC mol %	88.79
Country of origin	Denmark
Region of origin	Esbjerg W
Source of sample	Full-scale enriched biological phosphorus removal wastewater treatment plant
Geographical location	Esbjerg W
Latitude	55.488097
Longitude	8.430505
Depth	N/A
Altitude	N/A
Temperature of the sample	Mesophilic
pH of the sample	N/A
Relationship to oxygen	Facultative anaerobe
Energy metabolism	Potentially utilizing acetate and amino acids
Assembly	1 sample
Sequencing technology	Oxford Nanopore and Illumina Hiseq X
Binning software used	MetaBAT2
Assembly software used	CANU v1.8
Habitat	Full-scale enhanced biological phosphorus removal wastewater treatment plant
Miscellaneous, extraordinary features relevant for the description	N/A

Table S9. Protologues table for *Ca.* Azonexus amarohabitans.

Species name	*Candidatus* Azonexus amarohabitans
Genus name	*Candidatus* Azonexus
Specific epithet	amarohabitans
Type species of the genus	*Azonexus agitata* strain CKB
Genus status	Validly published
Species etymology	Description of *"Candidatus* Azonexus amarohabitans" sp. nov.: a.ma.ro.ha´bi.tans. G. fem. n. *amara*, trench, conduit, channel, here, a sewage conduit; L. part. adj. *habitans*, inhabitant; N.L. masch. adj. *amarohabitans*, pertaining to the habitat where the MAG was sequenced from.
Species status	sp. nov.
Designation of the type MAG	GCA_016709965.1
MAG/SAG accession number	GCA_016709965.1
Genome status	High-quality draft
Genome size	4174266
GC mol %	57.52
Country of origin	Denmark
Region of origin	Esbjerg W
Source of sample	Full-scale enriched biological phosphorus removal wastewater treatment plant
Geographical location	Esbjerg W
Latitude	55.488097
Longitude	8.430505
Depth	N/A
Altitude	N/A
Temperature of the sample	Mesophilic
pH of the sample	N/A
Relationship to oxygen	facultative anaerobe
Energy metabolism	Potentially utilizing acetate and amino acids
Assembly	1 sample
Sequencing technology	Oxford Nanopore and Illumina Hiseq X
Binning software used	MetaBAT2
Assembly software used	CANU v1.8
Habitat	Full-scale enhanced biological phosphorus removal wastewater treatment plant
Miscellaneous, extraordinary features relevant for the description	N/A

Table S10. Protologues table for *Ca.* Azonexus luticola.

Species name	*Candidatus* Azonexus luticola
Genus name	*Candidatus* Azonexus
Specific epithet	luticola
Type species of the genus	*Azonexus agitata* strain CKB
Genus status	Validly published
Species etymology	Description of *"Candidatus* Azonexus luticola" sp. nov.: lu.ti.vi´vens. L. neut. n. *lutum* mud, dirt; L. part. adj. *vivens*, living; N.L. masch. adj. *lutivivens*, pertaining to the habitat where the MAG was sequenced from.
Species status	sp. nov.
Designation of the type MAG	GCA_016705475.1
MAG/SAG accession number	GCA_016705475.1
Genome status	High-quality draft
Genome size	3578532
GC mol %	59.48
Country of origin	Denmark
Region of origin	Aalborg E
Source of sample	Full-scale enriched biological phosphorus removal wastewater treatment plant
Geographical location	Aalborg E
Latitude	57.045161
Longitude	10.045761
Depth	N/A
Altitude	N/A
Temperature of the sample	Mesophilic
pH of the sample	N/A
Relationship to oxygen	Facultative anaerobe
Energy metabolism	Potentially utilizing acetate and amino acids
Assembly	1 sample
Sequencing technology	Oxford Nanopore and Illumina Hiseq X
Binning software used	MetaBAT2
Assembly software used	CANU v1.8
Habitat	Full-scale enhanced biological phosphorus removal wastewater treatment plant
Miscellaneous, extraordinary features relevant for the description	N/A

References

Black, P.N., DiRusso, C.C., 2003. Transmembrane movement of exogenous long-chain fatty acids: proteins, enzymes, and vectorial esterification. Microbiol. Mol. Biol. Rev. 67, 454–472. https://doi.org/10.1128/MMBR.67.3.454-472.2003

Byrom, D., 1993. The synthesis and biodegradation of polyhydroxyalkanoates from bacteria. Int. Biodeterior. Biodegrad. 31, 199–208. https://doi.org/10.1016/0964-8305(93)90005-M

Chen, S., Zhou, Y., Chen, Y., Gu, J., 2018. fastp: an ultra-fast all-in-one FASTQ preprocessor. Bioinformatics 34, i884–i890. https://doi.org/10.1093/bioinformatics/bty560

Deng, Z.-L., Münch, P.C., Mreches, R., McHardy, A.C., 2022. Rapid and accurate identification of ribosomal RNA sequences via deep learning. Nucleic Acids Res. 50, e60–e60. https://doi.org/10.1093/nar/gkac112

Kopylova, E., Noé, L., Touzet, H., 2012. SortMeRNA: fast and accurate filtering of ribosomal RNAs in metatranscriptomic data. Bioinformatics 28, 3211–3217. https://doi.org/10.1093/bioinformatics/bts611

Singleton, C.M., Petriglieri, F., Wasmund, K., Nierychlo, M., Kondrotaite, Z., Petersen, J.F., Peces, M., Dueholm, M.S., Wagner, M., Nielsen, P.H., 2022. The novel genus, '*Candidatus* Phosphoribacter', previously identified as *Tetrasphaera*, is the dominant polyphosphate accumulating lineage in EBPR wastewater treatment plants worldwide. ISME J. 16, 1605–1616. https://doi.org/10.1038/s41396-022-01212-z

Tange, O., 2021. GNU SQL - A Command Line Tool for Accessing Different Databases Using DBURLs, ;login: The USENIX Magazine, April 2011:29-32

Wang, L., Yan, J., Wise, M.J., Liu, Q., Asenso, J., Huang, Y., Dai, S., Liu, Z., Du, Y., Tang, D., 2018. Distribution patterns of polyphosphate metabolism pathway and its relationships with bacterial durability and virulence. Front. Microbiol. 9, 782. https://doi.org/10.3389/fmicb.2018.00782

Zou, K., Huang, Y., Feng, B., Qing, T., Zhang, P., Chen, Y.-P., 2022. Cyanophycin granule polypeptide: a neglected high value-added biopolymer, synthesized in activated sludge on a large scale. Appl. Environ. Microbiol. 88, e00742-22. https://doi.org/10.1128/aem.00742-22
